# Sustainability of healthcare system improvements, programmes and interventions in acute care settings: protocol for a mixed methods systematic review

**DOI:** 10.1136/bmjopen-2024-094174

**Published:** 2025-02-07

**Authors:** Victoria Ramsden, Elizabeth McInnes, Peter Wilson, Franz E Babl, Lisa Kuhn, Julie Cowie, Pauline Campbell, Sandy Middleton, Catherine Wilson, Nicola Straiton, Emma Tavender

**Affiliations:** 1 Nursing Research Institute, St Vincent’s Health Network Sydney St Vincent’s Hospital Melbourne, Australian Catholic University, Australian Catholic University, Darlinghurst, New South Wales, Australia; 2 School of Nursing, Midwifery and Paramedicine, Australian Catholic University, North Sydney, New South Wales, Australia; 3 Healthy Brain and Mind Research Centre, Australian Catholic University, Fitzroy, Victoria, Australia; 4 Clinical Sciences, Murdoch Children's Research Institute, Parkville, Victoria, Australia; 5 Emergency Department, The Royal Children's Hospital Melbourne, Melbourne, Victoria, Australia; 6 Department of Paediatrics and Critical Care, The University of Melbourne - Parkville Campus, Melbourne, Victoria, Australia; 7 School of Nursing, Midwifery and Paramedicine, Australian Catholic University, Fitzroy, Victoria, Australia; 8 Yunus Centre for Social Business and Health, Glasgow Caledonian University, Glasgow, UK; 9 Department of Nursing and Community Health, School of Health and Life Sciences, Glasgow Caledonian University, Glasgow, UK

**Keywords:** Systematic Review, Emergency Departments, Implementation Science, Delivery of Health Care, Integrated, Quality in healthcare, Quality Improvement

## Abstract

**Abstract:**

**Introduction:**

Sustaining evidence-based care is challenging in all clinical settings. Acute care settings have a unique set of contextual factors that may impact sustainability (eg, fast-paced, regular staff turnover). Much of the previous research explores sustainability across undifferentiated healthcare settings making it difficult to determine factors that influence sustainability in acute care settings. The aim of this review is to identify facilitators and barriers that influence the delivery of sustained healthcare interventions (eg, integration of clinical guidelines) within adult and paediatric hospital-based acute care settings.

**Methods and analysis:**

A mixed methods systematic review updating Cowie *et al’s* (which included studies from 2008 to 2017) previously published systematic review will be conducted. The following databases will be searched: Medline, Embase, Cochrane Database of Systematic Reviews, CINAHL and Allied and Complementary Medicine (AMED), from November 2017 to the present for studies published in English. Relevant reference lists of included studies will be manually searched. Empirical quantitative and qualitative studies that report the sustainability of an intervention or programme in acute care settings using a theoretical framework(s), model(s) or theory(ies) to explore facilitators and barriers, will be included. Studies will be exported into Covidence (Melbourne) and pairs of reviewers will independently screen abstracts and full-text studies. The discussion will be used to resolve any disagreements and a third coauthor enlisted should a consensus not be reached. Two independent coauthors will extract key study characteristics and assess each study’s quality. Data will be extracted using Covidence (Melbourne). Evidence tables will be used to present descriptive data. Facilitators and barriers will be mapped to the Consolidated Framework for Sustainability Constructs in Healthcare and a narrative approach will be used to present key findings.

**Ethics and dissemination:**

No primary data will be collected so formal ethical approval is not required. Findings will be disseminated through peer-reviewed publications, presented at international conferences and on social media.

**PROSPERO registration number:**

PROSPERO CRD42024547535.

**STRENGTHS AND LIMITATIONS OF THIS STUDY**

- This systematic review will result in a better understanding of facilitators and barriers that influence the sustainability of healthcare interventions in acute care settings informing the behavioural change strategies likely to enhance future sustainability efforts.
- The review will be conducted in line with the Joanna Briggs Institute (JBI) methodology guidelines for mixed-method reviews.
- This protocol was developed in line with the Preferred Reporting Items for Systematic Reviews and Meta-Analysis Protocols guidelines.
- A lack of consensus on defining sustainability and a lack of consistent reporting on sustainability may hinder our review findings.
- The lack of recorded methodological detail regarding frameworks within abstracts may impact this review’s ability to capture some studies.

## Introduction

 Healthcare settings are complex, dynamic environments intended to provide high quality, safe and cost-effective evidence-based care to patients.^1 2^ There has been an increased focus on implementation of evidence-based practices in the last decade, yet even when successfully implemented, often the behaviour change and/or interventions are not sustained (eg, hand hygiene campaigns).^3^,^6^ Understanding the sustainability of evidence-based practices was identified as a research gap in the literature over a decade ago, however, it remains an under-researched area.^4^ Ensuring improvement efforts are successfully sustained safeguards against resources being wasted and poor patient outcomes as a result of receiving potentially harmful and/or unnecessary care.^7^

A universal definition of sustainability does not exist.^2^ Over the last 20 years, sustainability has been referred to as ‘routinisation’, ‘continuation’, ‘institutionalised’, ‘post-implementation’ and ‘maintenance’.^6 8^ Moreover, it is useful to distinguish between sustainability and sustainment which are often reported interchangeably.^9^ Sustainability is described as the magnitude that an evidence-based intervention, after the removal of external study support, can produce its intended benefits over a period of time.^9 10^ Sustainment however, is described as the continued use of the evidence-based intervention within routine practice.^9 10^

Evidence shows that sustaining evidence-based care is a challenge in any clinical setting.^11^,^14^ Previous research in healthcare settings has identified multiple factors influencing sustainability.^1 4 15^ Implementing and sustaining evidence-based practice interventions has an increased likelihood of success when the intervention is tailored to those who shape, deliver and participate in healthcare.^6^ Context variation between and within sites or departments may result in differing factors across healthcare settings.^1 4^ For example, acute care settings—where patients are treated for sudden, often emergent injuries or illnesses—present unique challenges due to their complex and dynamic nature compared with general medical wards.^16^ These environments are characterised by their fast pace, high staff turnover, large patient volumes and constant time pressures to address multiple and competing demands.^17^,^19^ Despite variations in healthcare settings, previous research predominantly examines factors influencing sustainability across undifferentiated hospital settings which complicates the identification of factors tailored to specific populations.^1 15^ Consequently, the lack of identified specific factors influencing sustainability in acute care settings impacts researchers’ and clinicians’ likelihood of sustaining, spreading and upscaling successful implementation interventions.^14^

Research suggests the use of frameworks, models or theories, to examine causal factors of sustainability is best practice,^4^ however, there is a lack of agreement about which framework is most effective.^20^ While a variety of frameworks exist to explicitly guide sustainability, the majority are designed for public health and community settings or are implementation frameworks that incorporate only some elements of sustainability.^4 21^ Penno *et al* identified three frameworks specific to acute care settings (Sustainability of Healthcare Innovations Framework,^22^ A Framework and a Measurement Instrument of Work Practice in long term care,^23^ and DCOM Framework with Realistic Evaluations.^24^,^4^ However, in some instances, it may be appropriate to consider using sustainability frameworks developed for a variety of hospital healthcare settings as these may provide alternative perspectives and lessons that are key to sustaining practice change.^4 7^

### Factors that influence sustained delivery of hospital-based healthcare interventions

Multiple systematic reviews have identified constructs and associated factors that may influence the sustained delivery of hospital-based healthcare interventions.^1 4 7 15^ Two of these four systematic reviews, identify existing sustainability frameworks/models/theories and relevant constructs to sustainability and healthcare setting.^4 7^ Lennox *et al* identified six specific constructs relevant to sustainability in any healthcare setting consisting of initiative design and delivery, negotiating initiative processes, resources, people involved, organisational setting and external environment.^7^ Penno *et al* identified seven constructs relevant to sustainability in acute care settings (innovation, adaptors, leadership and management, inner context, inner processes, outer context and outcomes), with four of the constructs aligning with Lennox *et al*’s findings and three adding to the literature.^4 7^

The third, more recently conducted, a systematic review of empirical studies, including 35 quantitative, 52 qualitative studies and 37 mixed methods studies, identified factors that had influenced the sustainability of improvement programmes in healthcare delivery systems.^15^ The most frequently reported factors were internal setting (leadership/support; staffing/turnover); and processes (training/supervision/support).^15^ However, factors were viewed on a continuum and therefore were not differentiated into either facilitators or barriers to sustainability (ie, policy could be a facilitator in one setting but a barrier in another).

The final systematic review by Cowie *et al* focused on empirical studies published prior to 2017 and examined factors influencing sustained delivery of a range of hospital-based interventions in healthcare settings in both adult and paediatric patients.^1^ The authors examined qualitative and mixed methods studies, conducted in any hospital-based healthcare setting for the whole of the hospital admission (ie, beyond the first 24 hours of presentation). The authors identified 32 studies, conducted in seven countries and eight healthcare settings, including four studies conducted in acute care settings. Factors influencing sustainability were mapped to the Consolidated Framework for Sustainability Constructs in Healthcare.^1 21^ They found similar factors that influenced sustainability in all hospital-based healthcare settings. Reported facilitators included clear accountability of roles and responsibilities during the initial implementation phase; strong leadership and champions; and adequate organisational support. The most frequently reported barrier was staff turnover. The authors’ findings reiterated that context and factors such as adequate investment in infrastructure which are specific to the organisational settings could have an impact on sustained intervention use.^1^

### Why is it important to do this review

Despite recognising differences in healthcare delivery and patient populations across settings, there remains a significant gap in understanding the factors that influence the sustainability of behaviour change and implementation interventions in acute care settings. Therefore, this systematic review, based on Cowie *et al’s* review, aims to synthesise the most current and relevant literature in this field.^1^ The review will make an important contribution to research applicable to clinical practice in acute care settings by identifying facilitators and barriers to sustaining healthcare interventions and programmes for both adult and paediatric populations. This knowledge will inform sustainability efforts undertaken by decision-makers, researchers, clinicians and healthcare professionals.

### Aims

Specific objectives of this mixed methods systematic review of empirical studies are to:

1. Identify, synthesise and appraise empirical studies that develop, test and use a theoretical framework to explore how healthcare interventions in any adult and paediatric clinical specialty acute hospital care settings are sustained, where the acute phase is defined as the first 24 hours of hospital visit/admission.
2. Identify facilitators and barriers that influence the delivery of sustained healthcare interventions in hospital-based acute care settings.

## Methods and analysis

### Design

We will conduct a systematic review of quantitative, qualitative and mixed-methods studies including quality improvement studies in line with the Joanna Briggs Institute (JBI) methodological guidance for mixed-methods reviews.^25^ We will report data in accordance with the Preferred Reporting Items for Systematic Reviews and Meta-Analysis (PRISMA) statement^26^ and the current protocol has been developed using the PRISMA Protocol checklist (online supplemental 1).^27^ This study was registered with the International Prospective Registry of Systematic Reviews (PROSPERO) (CRD42024547535). The protocol start date was 7 September 2024 and the systematic review is expected to conclude by 11 November 2025.

### Key definitions

To assist with the application of the study criteria, the following operational definitions will be used.

#### Sustainability

In the past decade, despite considerable effort, a single definition of sustainability is still not available. Moore *et al* identified four syntheses of sustainability. Of the 209 articles identified, only 24 (11.5%) included a definition of sustainability.^28^ These definitions were used by Moore *et al* to create an amended definition with five key constructs which incorporate both concepts of sustainability (continued clinician behaviour change) and sustainment (continued use of the intervention strategy) (table 1).^28^ To align with Cowie *et al*’s original review and the larger body of work on sustainability, Moore *et al*’s definition will be used.^1 28^

**Table 1:** The five constructs of Moore *et al’s* sustainability definition^28^

1	After a defined period of time
2	Intervention/programme has continued to be delivered
3	Behaviour change is maintained
4	Intervention/programme and/or behaviour change may adapt and/or evolution
5	Continued individual/system benefits

#### Acute care settings

This systematic review is part of a larger sustainability study which explores whether improved adherence to evidence-based care is sustained within acute care settings. For consistency, we have aligned our definition of acute care in line with this larger sustainability study, whereby acute care is defined as any hospital-based care an adult or paediatric patient receives within the first 24 hours of their hospital visit, including emergency department visits and ward admissions.

#### Healthcare intervention

A healthcare intervention is defined as any intervention or programme that is delivered in a hospital environment (emergency departments and inpatient areas) within the first 24 hours of care.^29^ The intervention must be delivered or received by staff and/or carers/patients (directly or indirectly) in any clinical specialty and be aimed at improving patient care. Any intervention or programme that is provided in ambulatory care, is virtual or laboratory-based, will be excluded.

#### Framework

Within implementation science, the terms framework, model and theory are often used interchangeably.^30^ Consistent with Cowie *et al*, we refer to frameworks, models and theories collectively as a framework. We will also use Nilsen’s taxonomy of frameworks, models and theories that define a framework as an overview or structure that aims to pinpoint factors which influence the outcomes of implementation.^30 31^

#### Facilitators and barriers

Our definitions of facilitator and barrier are consistent with those proposed by Bach-Mortensen *et al*.^32^ We define any factor that contributes to the sustainability of intervention beyond the implementation period as a facilitator (eg, skilled clinical champions). Any factor that obstructs the sustained delivery of an intervention will be defined as a barrier (eg, lack of resources).^1 7^

### Eligibility criteria

Eligible papers will include empirical studies published in English, relating to the sustainability of a healthcare intervention or programme conducted in adult and paediatric hospital acute care settings. In line with our definitions of healthcare interventions and acute care settings, included studies will need to focus on the sustainability of evidence-based healthcare interventions intended to improve patient (adult and paediatric) care within the first 24 hours of presentation to a hospital in any clinical specialty. Studies that use a structured approach to explore facilitators and barriers will be included (ie, used a framework, model or theory to identify factors that influenced sustainability). As Cowie and colleagues completed the search in December 2017,^1^ we will include papers published after 1 November 2017 until the present (table 2).

**Table 2:** Research strategy containing inclusion and exclusion criteria

	Inclusion criteria	Exclusion criteria
Population/setting	Patients of any age (adults or paediatric) who present to a hospital in the first 24 hours for either physical or mental health reasons.	Any study not conducted in hospital acute care settings. Any study performed across multiple settings, when results relating to the acute care setting are not clearly identifiable.
Intervention/treatment	Any empirical study that specifically aims to provide a sustainable intervention delivered or received by staff and/or patients/carers to improve patient care within the first 24 hours in a hospital-based setting.	Any study that does not discuss a specific programme or intervention. A service provided that is regarded as prehospital, outpatients, ambulatory care, GP-led clinical practice, virtual or lab-based interventions (eg, first responders).
Comparators	We will include all study types, with and without comparators.	Nil
Outcome	Primary outcome:Establishing facilitators and barriers to sustaining the delivery of healthcare intervention in the first 24 hours of presentation or admission to adult or paediatric acute care settings.Secondary:Comprehensiveness of reported sustainability assessed per Moore *et al’s* definition of sustainability.^28^Identification of frameworks employed to explore facilitators and barriers.Identification of the category of framework employed mapped to Nilsen’s taxonomy.^30^Determining the level of framework visibility mapped to Bradbury-Jones *et al’s* typology.^34^	Sustainability is not the specific concern of the study (ie, focused on initial implementation of programme/intervention).No reference is made to sustainability frameworks, theories or models.
Research design	Empirical studies including qualitative, quantitative and mixed methods studies, relating to sustainability of a healthcare intervention or programme conducted in adult or paediatric hospital acute care settings (within the first 24 hours of presentation).Quantitative studies will include randomised controlled trials (RCTs), quasi-randomised trials, controlled before-and-after (CBA) studies. Non-randomised studies including non-randomised controlled trials, cohort studies, CBA studies and historically controlled studies; and retrospective or prospective cohort studies that include a control group.Qualitative papers will comprise any study designs including, but not limited to, studies using phenomenology, grounded theory, ethnography, action research and descriptive research.	Secondary research including systematic reviews will be excluded, however, reference lists of related systematic reviews will be reviewed for missed and potentially relevant papers. Any studies that are unpublished, not peer-reviewed publications or which contain non-research study designs (eg, unstructured reviews or overviews, theoretical papers, commentaries or opinion papers, protocol, case studies, editorials, audits, letters, dissertations and conference abstracts).
Search limitsLanguage	English	
Publication date	1 November 2017 to present	

GPgeneral practitioner

Studies that do not report using a theoretical framework/model/theory will be excluded as will those that report interventions aimed at improving care beyond the first 24 hours of hospital visit/admission.

### Information sources and search strategy

#### Search strategy

The following process will be used to develop search terms. An information specialist will revise the original Cowie *et al’s* systematic review search to update search terms and increase sensitivity for the new search.^1^ We will examine the search strategies and terms that are published in high-quality empirical studies on sustainability to further refine the search strategy. Key terms will then be combined using a series of free text terms and Medical Subject Headings for (1) sustainability (eg, duration, long term) (2) framework (eg, frameworks, models, theories) and (3) acute care (emergency medicine). Finally, pilot searches will be conducted using the Medline database (Ovid) and the search terms refined iteratively prior to use with other databases.

The search strategy will be adapted for each database, Boolean operators, truncations and wildcards will be used to account for variations across databases. Table 3 outlines our search terms.

**Table 3:** Search strategy

Sustainability	Sustain* OR implement* OR ‘long-term implement*’ OR discontin* OR deadoption OR ‘capacity building’ OR institutationali$ation OR ‘knowledge utili$ation’ OR institutionali?tion OR durabil* OR maintenance OR routini?ation OR continua* OR resilien* OR viab* OR stability OR stable OR persist* OR adhere* OR ‘dis-investment’ OR disinvestment OR ‘de-implementation’ OR deimplementationMeSH Terms:(MH ‘Implementation Science) OR (MH ‘Delivery of Health care)
AND	
Frameworks	Theor* OR model* OR principle* OR construct* OR framework*MeSH Terms:(MH ‘Frameworks’)
AND	
Acute Care	‘Acute care’ OR ‘acute ward’ Or ‘acute hospital’ OR ‘emergency care’ OR ‘emergency medicine’ OR ‘emergency department’ OR ED OR ‘emergency nursing’ OR ‘emergency ward’ OR ‘accident emergency’ OR ER OR inpatient OR ‘in-patient’ OR ward* OR unit* OR hospital* OR ‘Critical Care’MeSH Terms: (MH ‘Patient Care’) OR (MH ‘Patient Admission’) OR (MH ‘Hospitals’) OR (MH ‘Inpatients’) OR (MH ‘Critical Care’) OR (MH ‘Emergency Medicine’) OR (MH ‘Emergency Service’) OR (MH ‘Emergency Nursing’) OR (MH ‘Trauma Centres’) OR (MH ‘Emergency Medical Services’)
AND	
Intervention	Intervent* OR program* OR evaluat* OR facilitat* OR barrier*MeSH Terms:(MH ‘Quality assurance’) OR (MH ‘Health Care) OR (MH ‘Quality Improvement’) OR (MH ‘Program Evaluation’)

#### Electronic searches

The selection of healthcare databases, search terms and strategy and the initial search will be made in consultation with an information search strategy specialist. We will systematically search the following electronic databases from 1 November 2017 to present:^1^ Medline; Embase; CENTRAL databases (including Cochrane Database of Systematic Reviews); CINAHL (EBSCO) and Allied and Complementary Medicine (AMED) (Ovid).

#### Other searches

The reference lists from included papers will be examined to capture relevant studies not discovered by the database searches and will be independently screened by two coauthors for potential relevance.

### Study records

#### Data management

All identified papers will be entered into the EndNote platform for de-duplication and then imported into Covidence (Melbourne) for screening.

#### Study selection

Stage 1: Two coauthors will independently screen all titles and abstracts of papers identified in the electronic databases and any additional papers identified in the manual search of reference lists against the inclusion and exclusion criteria, removing any clearly irrelevant papers. Studies ranked as irrelevant by both coauthors will be excluded. Any disagreements will be discussed and a third coauthor will be enlisted should a consensus not be reached.

Stage 2: The full-text papers for the remaining studies will be obtained and two coauthors will then independently assess these against the selection criteria. Disagreements will be resolved initially through discussion, followed by adjudication by a third independent coauthor as required.

### Data extraction items

A data extraction form will be created and piloted to extract the data in table 4.

**Table 4:** Data to be included in data extraction form

Data extraction component	Explanation
Study characteristics	Author
	Date of publication
	Country
	Aims
	Study design
Study population	Targeted population
Participant demographics	Sample size
	Patient group
	Details of healthcare professional staffing groups involved including any data on their length of service, job role
Study setting and other relevant contextual information	Intervention/programme aimsDetails of the intervention/programme
Theoretical frameworks	Including justification for framework· Category of implementation framework as per Nilsen’s taxonomy:^30^ Process models Determinant frameworks Classic theories Implementation theories or Evaluation frameworks· Theoretical visibility as Bradbury-Jones *et al*.^34^ Level 1 seemingly absent Level 2 implied Level 3 applied partially Level 4 applied retrospectively Level 5 applied consistently
Comparison conditions	Conditions used to compare against intervention
Details of the intervention and interventionist	Template for intervention description and replication guidelines (TIDieR):^43^ Brief name of intervention Why—rationale for the intervention What materials and procedures were used Who provided the interventions How was the intervention delivered Where was the intervention delivered When was the intervention delivered and how often Tailoring, was the intervention planned to be personalised Where modifications made to the interventions How well was the intervention delivered, that is, was there a plan to assess intervention adherence and fidelity and did it occur?
Outcome measures	Primary outcome:Facilitators and barriers to sustainability explored using a framework, model or theory.Secondary outcomes: Identify comprehensiveness of reported sustainability—mapped to five constructs of Moore *et al’s* sustainability:^28^ After defined period of time (eg,>6 months) Intervention continues to be delivered and/or (at least on component or more) Intended individual behaviour change is maintained Both (2) or (3) may evolve or adapt Continues to produce beneficial outcomes (individual or system) Identification of frameworks employed to explore facilitators and barriers (1) Name Category of implementation framework as per Nilsen’s taxonomy^30^ Theoretical visibility as per Bradbury-Jones et al^34^
Key findings	Study outcomes Number of studies using framework, model or theory to explore sustainability Which framework, model or theory has been used to explore sustainability Whether sustainability occurred and the time periods over which this was measured Other relevant findings

While the definition of sustainability is broad, we acknowledge that it may be hard to operationalise all key concepts, especially if the information on each is not fully reported in the study (ie, after a defined period). Therefore, following Cowie *et al’s* lead, eligible studies will be judged by the review team against each of Moore *et al’s* five constructs to document any missing information, highlight any gaps in the evidence and report sustainability and sustainment separately.^1 28 31^

Data from eligible publications will be extracted into Covidence (Melbourne) by two independent coauthors. Any discrepancies in data extraction will be resolved through discussion between coauthors and referred to a third coauthor for adjudication if consensus cannot be reached.

#### Data collection and analysis

##### Coding

To date, there is no single comprehensive tool that can be used to identify and code facilitators and barriers for sustained long-term interventions. As such, the Consolidated Framework for Sustainability Constructs in Healthcare which provides a predefined list of 40 constructs for sustainability categorised under six emergent domains ((1) The external environment; (2) Initiative design and delivery; (3) Negotiating the initiative processes; (4) Organisational setting; (5) The people involved; (6) Resources) will be used to code the facilitators and barriers.^7^ In line with our definition of acute care settings, this framework was chosen as it was previously suggested to provide the most useful insight into sustainability in any hospital setting and demonstrated it could be used to explore facilitators and barriers in all hospital settings including acute care settings.^31^ Additionally, it was chosen as Penno *et al*, found that four Consolidated Framework for Sustainability Constructs in Healthcare domains aligned with the seven constructs, they deemed necessary to evaluate the sustained use of evidence-based practices within acute care settings.^4^

A deductive and inductive approach to identifying factors influencing sustainability will be used. NVivo V.12 qualitative data analysis software will be used to complete the analysis.^33^ The deductive approach will involve coding to the 40 predefined lists of constructs of the Consolidated Framework for Sustainability Constructs in Healthcare.^7^ Two independent coauthors will extract data identified as important in the delivery of healthcare interventions in acute care areas (author, year, country, direct quotes, page numbers). Data will be categorised as either facilitators or barriers or neutral, coding will be guided by Lennox *et al’s* breakdown of the framework which includes a detailed description, definitions and examples of the predefined constructs.^7^ Facilitators and barriers that cannot be categorised using the predefined constructs will be coded as ‘other’ and an inductive coding approach will be used to develop themes and subthemes of these additional data. Any differences will be resolved through discussion with other research team members.

Two independent coauthors will extract data on the comprehensiveness of sustainability by mapping included studies against each of Moore *et al’s* five construct definitions of sustainability.^28^ Two independent coauthors will also extract data on the identification of the framework used in the included studies, the framework category mapped to Nilsen’s taxonomy and framework visibility mapped to Bradbury-Jones *et al’s* typology.^30 34^ Any differences will be resolved through discussion with other research team members.

### Outcomes and priorities

Outcomes are listed in table 4 with the primary outcome being facilitators and barriers to sustainability. Studies must incorporate the use of a framework to explore factors.

### Risk of bias in individual studies

Appropriate quality tools will be used to assess the quality and risk of bias of included studies. The appropriate Critical Appraisal Skills Programme tools will be used to assess quantitative and qualitative studies, the Mixed Method Appraisal Tool will be used to assess mixed methods studies and the Standards for Quality Improvement Reporting Excellence will be used to assess quality improvement studies.^35^,^37^ This process will be undertaken by two independent coauthors, when disagreements occur, attempts to reach a resolution will occur initially through conversation, followed by deliberation by a third coauthor as required.^38^

### Data synthesis

Evidence tables will be used to present descriptive data such as year, country and other background factors. They will include data from newly identified studies and relevant studies from Cowie *et al’s* systematic review.^1^ We do not plan to conduct a meta-analysis due to the expected large heterogeneity of study designs which is similar to previous systematic reviews of this type.^6 31 39 40^ This is a mixed-methods review, that will use a convergent integrated approach to synthesise the quantitative and qualitative data.^41^ A narrative approach will be used to present quantitative and qualitative findings. Qualitative data will be analysed by themes, grouping facilitators and barriers providing tabular and narrative summaries of the key characteristics of the facilitators and barriers that influence the delivery of sustained healthcare intervention. Quantitative data will also provide information on the frequency of factors reported.

### Meta-biases

We will reduce publication bias and selective outcome reporting by publishing this protocol a priori, providing a detailed overview of our search rationale, strategy and inclusion criteria.^28 38^ By providing detailed inclusion, exclusion and search criteria and a data extraction form that will be used independently by separate authors to screen and extract data, we aim to avoid selection bias.^42^

### Ethics and dissemination

As this is a review of published literature, no ethics approval was required. Findings will be disseminated via publication or conferences. Protocol revisions will be documented and updated on PROSPERO.

## supplementary material

10.1136/bmjopen-2024-094174 online supplemental file 1
