# Supplementary material for: Sustainability of healthcare system improvements, programmes and interventions in acute care settings: protocol for a mixed methods systematic review
Source: BMJ Open. 2025 Feb 7;15(2):e094174. doi: 10.1136/bmjopen-2024-094174 (PMC11808872; doi:10.1136/bmjopen-2024-094174)
Supplement: Supplementary file 2 [file bmjopen-2024-094174.reviewer_comments.pdf]

PEER REVIEW HISTORY

BMJ Open publishes all reviews undertaken for accepted manuscripts. Reviewers are asked to complete a checklist review form and are provided with free text boxes to elaborate on their assessment. These free text comments are reproduced below.

ARTICLE DETAILS

Title (Provisional)

The sustainability of healthcare system improvements, programmes and interventions in acute care settings: Protocol for a mixed methods systematic review

Authors

Ramsden, Victoria; McInnes, Elizabeth; Wilson, Peter; Babl, Franz E; Kuhn, Lisa; Cowie, Julie; Campbell, Pauline; Middleton, Sandy; Wilson, Catherine; Straiton, Nicola; Tavender, Emma

---

VERSION 1 - REVIEW

**Reviewer**	**1**
**Name**	**Kaya, Murtaza**
**Affiliation DEPARTMENT**	**Kutahya Saglik Bilimleri Universitesi, EMERGENCY**
**Date**	**24-Nov-2024**
**COI**	**None**

Dear Authors

I think the manuscript is well-written, and the research question is both clear and relevant to the field of acute care sustainability. The methodology is robust, and the study objectives align well with the chosen study design. This protocol has the potential to make a significant contribution to the understanding of sustainability in healthcare interventions.

However, I have a few minor suggestions that could strengthen the manuscript further:

Contextual Factors: Please expand on how contextual factors within diverse acute care settings will be analyzed. A more detailed description of this process would enhance the applicability of the findings to various healthcare environments.

Data Synthesis: Provide additional clarity on the approach to synthesizing qualitative and quantitative data. For example, specifying how the narrative synthesis and thematic coding will be integrated could improve the transparency and reproducibility of the study.

Addressing these points will further improve the overall clarity and rigor of the protocol. I commend the authors for their comprehensive and thoughtful approach to this important area of research.

Reviewer 2

Name Altaye, Kassaye

Affiliation University of Gondar College of Medicine and Health Sciences, Emergency and Critical Care Medicine

Date 01-Dec-2024

COI None

It is well written article in compliance with PRISMA-P 20215 and 2020 update.

Reviewer 3

Name ouellet, simon

Affiliation Université du Québec à Rimouski, Department of Health Sciences

Date 17-Dec-2024

COI None

The sustainability of healthcare system improvements, programmes and interventions in acute care settings: Protocol for a mixed methods systematic review

ABSTRACT:

Line 113: Sustained healthcare interventions

- Would it be possible to provide examples of such interventions? (e.g., continuous training programs, quality improvement initiatives, integration of clinical guidelines).

Line 121-124: Empirical quantitative and qualitative studies that report sustainability of an intervention or programme in acute care **settings and use a structured approach (i.e., those that report using theoretical framework(s), model(s) or theory(ies))** to explore facilitators and barriers, will be included.

- Does this mean that studies not using a theoretical model will be excluded? If so, consider stating this more explicitly.

INTRODUCTION:

Line 154-155: yet even when successfully implemented, often the behaviour change and/or interventions are not sustained

- Would it be possible to provide examples of such interventions? (e.g., hand hygiene campaigns, patient safety checklists)

Line 192-193: Whilst, Penno et al.'s identified three frameworks specific to acute care settings…

- It would be helpful to explicitly name these three frameworks and provide their specific references here, rather than grouping them at the end of the text.

METHODS AND ANALYSIS

Line 364-367: Stage 1: Two co-authors will independently screen all titles and abstracts of papers identified in the electronic databases and any additional papers identified in the manual search of reference lists against the inclusion and exclusion criteria, removing any clearly irrelevant papers. Studies ranked as irrelevant by both co-authors will be excluded.

- Will a third reviewer be involved in cases of disagreement between the two coauthors? If so, consider mentioning this (As mentioned in Stage 2).

Line 394-395: As such, the Consolidated Framework for Sustainability Constructs in Healthcare, which provides a predefined list of 40 constructs for sustainability categorised under six emergent domains.

- It would be beneficial to list these six domains for greater clarity.

Line 425-426: Outcomes are listed in Table 4. Studies that explore the factors which influence sustainability must incorporate the use of a theory, model or framework

- It would be helpful to mention the key outcomes in the text and refer to the table for further details.

CONCLUSION

Line 471-472 : Findings will inform future sustainability strategies of acute healthcare interventions.

- The conclusion could be strengthened by being more explicit, emphasizing the importance of your review in guiding the development of targeted and evidencebased strategies for sustaining acute healthcare interventions. This would highlight the value of your work in shaping future practices.

TABLES

Table 2: Comparators

- Will studies without comparators be excluded? If so, please specify this in the table.

Table 4: Excellent table overall! Good job.

- Consider adding examples of the key findings being sought. **STUDY LIMITATIONS (missing)**
- Although there is a section on Risk of Bias in Individual Studies and Meta-biases, it would be pertinent to add a section on the limitations of the study. This would provide a more comprehensive understanding of the study's scope and potential constraints.

Happy Holidays

VERSION 1 - AUTHOR RESPONSE

**Reviewer 1 (Dr. Murtaza Kaya) comments, with author responses below:**
1. *Contextual Factors: Please expand on how contextual factors within diverse acute care settings will be analyzed. A more detailed description of this process would enhance the applicability of the findings to various healthcare environments.*<br><br>*Authors response:*<br>*This comment was not actioned. A systematic review by Rogers et al (2020) titled 'Defining and assessing context in healthcare implementation studies' recommends that "Due to the variability in how context is assessed, it is recommended that a standardised approach using qualitative methods informed by a comprehensive framework is the most suitable assessment to explore the complexity of this phenomenon". Based on this recommendation we chose the Consolidated Framework for Sustainability Constructs in Healthcare to explore contextual factors. We will however extract as much contextual detail as we can from the studies and consider these factors when reporting and analysing the results in our final review paper.*
2. Data Synthesis: Provide additional clarity on the approach to synthesizing qualitative and quantitative data. For example, specifying how the narrative synthesis and thematic coding will be integrated could improve the transparency and reproducibility of the study.<br><br>*Authors response:*<br>*Further clarification on the approach to synthesizing quantitative and qualitative data has been provided to the data synthesis section. Refer to lines 472 – 482.*
**Reviewer 2 (Dr. Kassaye Altaye) comments, with author responses below:**
1. It is well written article in compliance with PRISMA-P 20215 and 2020 update.<br><br>*Thank you - no author response needed.*
**Reviewer 3 (Dr. Simon Ouellet) comments, with author responses below:**
1. Line 113: Sustained healthcare interventions:<br>• Would it be possible to provide examples of such interventions? (e.g., continuous training programs, quality improvement initiatives, integration of clinical guidelines).<br><br>*Authors Response:*<br>*Completed. Refer to line 111.*
2. Line 121-124: Empirical quantitative and qualitative studies that report sustainability of an intervention or programme in acute care settings and use a structured approach (i.e.,

those that report using theoretical framework(s), model(s) or theory(ies)) to explore facilitators and barriers, will be included.<br>• Does this mean that studies not using a theoretical model will be excluded? If so, consider stating this more explicitly.<br><br>*Authors Response:*<br>*Yes, the above sentence does mean that studies not using a theoretical model will be excluded. This has been clarified to state this more explicitly. Refer to line 121.*
3. Line 154-155: yet even when successfully implemented, often the behaviour change and/or interventions are not sustained<br>• Would it be possible to provide examples of such interventions? (e.g., hand hygiene campaigns, patient safety checklists)<br><br>*Authors Response:*<br>*Example provided as requested. Refer to line 173-174.*
4. Line 192-193: Whilst, Penno et al.'s identified three frameworks specific to acute care settings…<br>• It would be helpful to explicitly name these three frameworks and provide their specific references here, rather than grouping them at the end of the text.<br><br>*Authors Response:*<br>*This sentence has been revised to include the three frameworks identified by Penno have been explicitly named. Refer to lines 211-213.*
5. Line 364-367: Stage 1: Two co-authors will independently screen all titles and abstracts of papers identified in the electronic databases and any additional papers identified in the manual search of reference lists against the inclusion and exclusion criteria, removing any clearly irrelevant papers. Studies ranked as irrelevant by both co-authors will be excluded.<br>• Will a third reviewer be involved in cases of disagreement between the two co-authors? If so, consider mentioning this (As mentioned in Stage 2).<br><br>*Authors Response:*<br>*A third reviewer will be involved in cases of disagreements. This has been added to stage 1, as suggested. Refer to line 394-395.*
6. Line 394-395: As such, the Consolidated Framework for Sustainability Constructs in Healthcare, which provides a predefined list of 40 constructs for sustainability categorised under six emergent domains.<br>• It would be beneficial to list these six domains for greater clarity.<br><br>*Authors Response:*<br>*This sentence has been revised to list the Consolidated Framework for Sustainability Constructs in Healthcare six domains. Refer to lines 423 - 425.*
7. Line 425-426: Outcomes are listed in Table 4. Studies that explore the factors which influence sustainability must incorporate the use of a theory, model or framework<br>• It would be helpful to mention the key outcomes in the text and refer to the table for further details.<br><br>*Authors Response:*<br>*We have now listed the primary outcome measure in the text with more detail provided in Table 4. Refer to line 455-456.*
8. Line 471-472: Findings will inform future sustainability strategies of acute healthcare interventions.

• The conclusion could be strengthened by being more explicit, emphasizing the importance of your review in guiding the development of targeted and evidence- based strategies for sustaining acute healthcare interventions. This would highlight the value of your work in shaping future practices.<br><br>*Authors Response:*<br>*This comment was not actioned as the conclusion was removed as per the editor's comment but we will take it on board for the conclusion of our final review paper.*
9. Table 2: Comparators<br>• Will studies without comparators be excluded? If so, please specify this in the table.<br><br>*Authors Response:*<br>*Studies with and without comparators will be included. This has been clarified in Table 2. Refer to Table 2.*
10. Table 4: Excellent table overall! Good job.<br>• Consider adding examples of the key findings being sought.<br><br>*Authors Response:*<br>*An example was added to the key findings being sought as suggested. Refer to key findings in Table 4.*
11. STUDY LIMITATIONS (missing)<br>• Although there is a section on Risk of Bias in Individual Studies and Meta-biases, it would be pertinent to add a section on the limitations of the study. This would provide a more comprehensive understanding of the study's scope and potential constraints.<br><br>*Authors Response:*<br>*This protocol was guided by the PRISMA -P checklist. A study limitations section was not included as this was not a required section on this checklist. As this is a protocol, we only have a limited understanding of the study's scope and potential constraints. As such we have identified potential study limitations in the strengths and limitations section, after the abstract. We plan to provide a more comprehensive discussion of our identified study limitations in our completed review discussion section.*
